# Supplementary material for: Eyewire II – A connectomic resource for resolving cell types and circuits of the mouse retina
Source: bioRxiv. 2026 Jun 1:2026.05.28.727403. Preprint. [Version 1] doi: 10.64898/2026.05.28.727403 (PMC13252113; doi:10.64898/2026.05.28.727403)
Supplement: Supplement 1 [file NIHPP2026.05.28.727403v1-supplement-1.pdf]

bioRxiv preprint doi: https://doi.org/10.64898/2026.05.28.727403; this version posted June 1, 2026. The copyright holder for this preprint (which was not certified by peer review) is the author/funder, who has granted bioRxiv a license to display the preprint in perpetuity. It is made available under a CC-BY 4.0 International license.

Supplementary Note 1: Imaged EM stack

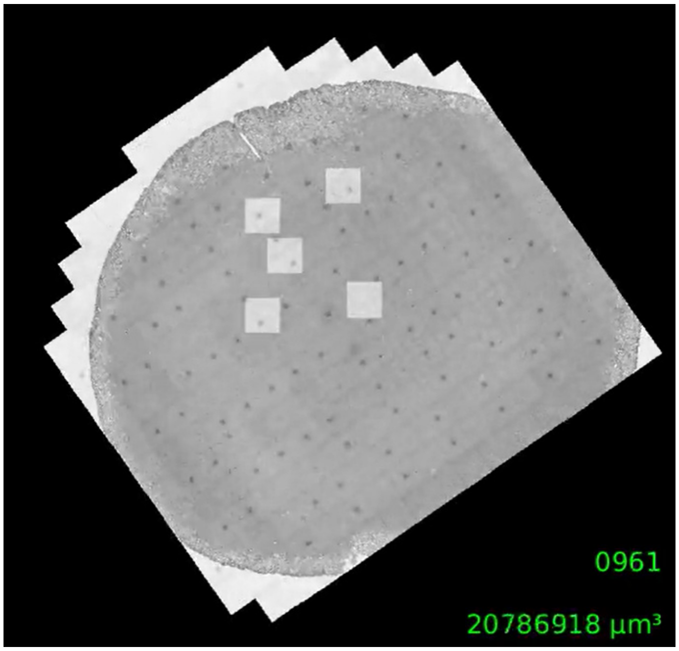

Figure S1. Supplement 1 to Fig. 1 – Movie of imaged EM stack. See Movie-figure1-supplemental-1.mp4.

bioRxiv preprint doi: https://doi.org/10.64898/2026.05.28.727403; this version posted June 1, 2026. The copyright holder for this preprint (which was not certified by peer review) is the author/funder, who has granted bioRxiv a license to display the preprint in perpetuity. It is made available under a CC-BY 4.0 International license.

Supplementary Note 2: BC ribbon count and size distributions

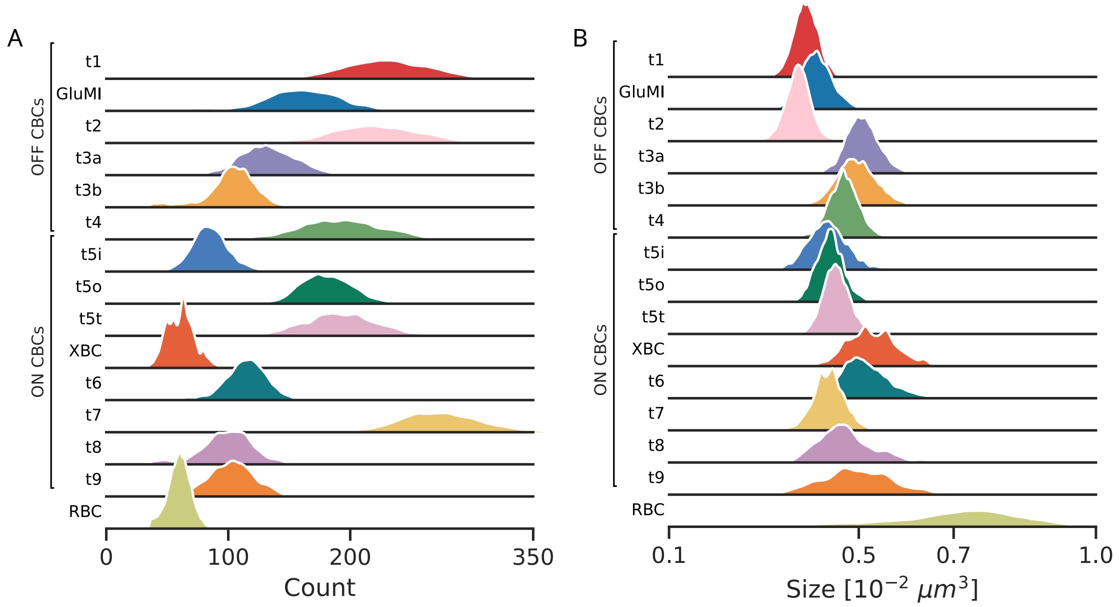

Figure S2. Supplement 1 to Fig. 8 – BC ribbon count and size distributions. (**A**) Normalized distributions of ribbon count per cell for each BC type. (**B**) Normalized distributions of mean ribbon size per cell for each BC type. Same dataset and BC type colors as in Fig. 8F and Fig. 4.

bioRxiv preprint doi: https://doi.org/10.64898/2026.05.28.727403; this version posted June 1, 2026. The copyright holder for this preprint (which was not certified by peer review) is the author/funder, who has granted bioRxiv a license to display the preprint in perpetuity. It is made available under a CC-BY 4.0 International license.

Supplementary Note 3: BC ribbon statistics

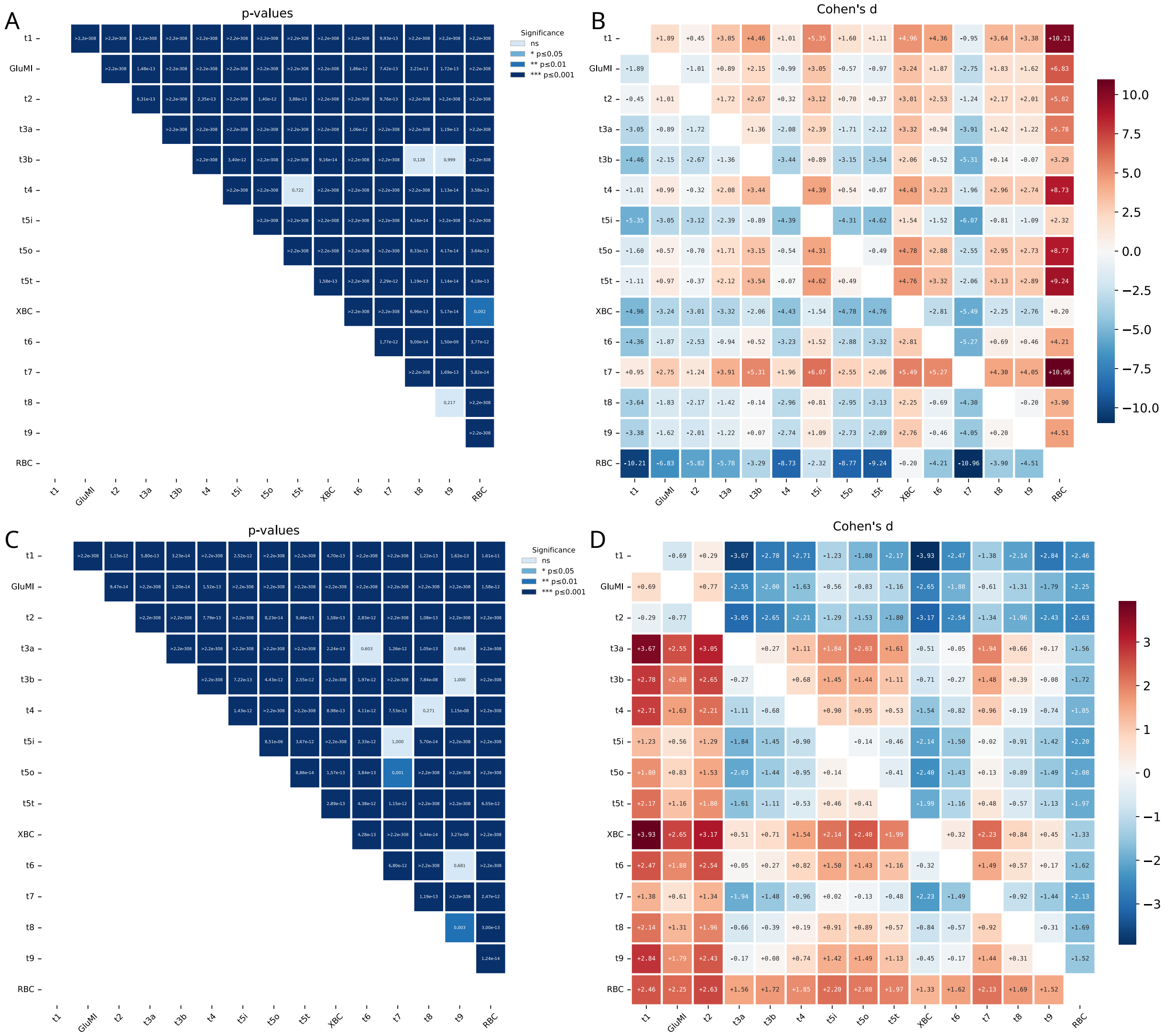

Figure S3. Supplement 2 to Fig. 8 – BC ribbon statistics. (**A,B**) p-values ((A), post-hoc pairwise Games-Howell test) and effect size ((B), Cohen's d) for ribbon counts. (**C,D**) Same as in (A,B) but for mean ribbon size. Same dataset and BC type colors as in Fig. 8F and Fig. 4.
